# Is it safe to use a ureteral access sheath in an unstented ureter?

**DOI:** 10.1186/s12894-019-0509-x

**Published:** 2019-08-29

**Authors:** Asaf Shvero, Haim Herzberg, Dorit Zilberman, Yoram Mor, Harry Winkler, Nir Kleinmann

**Affiliations:** 1 Department of Urology, The “Chaim Sheba” Medical Center, Ramat-Gan, Israel. Affiliated to Sackler School of Medicine, Tel-Aviv University, Tel-Aviv, Israel; 2 Department Of Urology, Sourasky Medical Center, Tel-Aviv, Israel. Affiliated to Sackler School of Medicine, Tel-Aviv University, Tel-Aviv, Israel

**Keywords:** Ureteroscopy, Ureter, Stricture, Lithotripsy, Ureteral access sheath

## Abstract

**Background:**

The aim of this study was to examine ureteral stricture rate after the use of UAS in an unstented ureter and compare complications of smaller vs. larger-caliber UAS.

**Methods:**

We conducted a retrospective analysis of consecutive RIRS for renal stones, with the use of UAS in unstented ureters. We excluded cases with previous ureteroscopies, who carried ureteral stent or nephrostomy, had impacted stones, underwent radiation treatment, or had urinary tract malignancies. The primary outcome was formation of ureteral strictures diagnosed by hydronephrosis in ultrasound test and late secretion in dynamic renal scan. Secondary outcome was stone-free-rate (SFR) and complications. In addition, we compared safety and efficacy of smaller (9.5/11.5Fr) vs. larger-caliber (12/14Fr) UAS.

**Results:**

The cohort included 165 patients with a median follow-up time of 115 days. There was no case of ureteral stricture formation after the use us UAS, despite using a larger-caliber UAS in nearly half the cases. Larger-caliber UAS was not associated with more complications compared to the smaller-caliber one (*p* = 0.780). SFR was non-significantly higher in the larger-caliber UAS group (*p* = 0.056), despite having a larger stone burden, and only stone number was associated with SFR (*p* = 0.003).

**Conclusions:**

These data suggest that the use of UAS during RIRS in an unstented ureter is safe and does not involve ureteral stricture formation after one procedure. Furthermore, the use of wider sheaths was not found to be associated with higher complications rate.

## Background

The role of retrograde intra-renal surgery (RIRS) in stone disease is continuously increasing due to improved surgical outcomes compared to extracorporal shockwave lithotripsy [1] and lower complication rates compared to percutaneous nephrolithotomy [2].

Ureteral access sheaths (UASs) are being commonly used during RIRS in order to decrease intra-renal pressure, improve visibility, and provide easy access to the renal pelvico-calyceal system; consequently, reducing the risk of bleeding and infections. Larger-diameter UASs provide improved visibility and facilitates the removal of larger stone fragments [3–6].

However, several studies report that the use of UAS might cause severe damage to the ureteral wall, tissue ischemia with subsequent reperfusion damage, and benign ureteral strictures [5, 7, 8]. The clinical significance of this damage to the ureter still remains controversial [9]. Nonetheless, these reports have still led many urologists to avoid the use of UAS in unstented ureters due to fear of associated ureteral strictures. Furthermore, even when they eventually use UAS in a virgin ureter, some urologists avoid using large-caliber UAS. Ureteral strictures following ureteroscopy were reported to develop in a short period of time after surgery, mostly during 4 weeks of follow-up [10].

The objective of this study was to determine the rate of clinically significant ureteral strictures following the use of ureteral access sheaths in an unstented ureter during RIRS, and to compare complications of smaller and larger-diameter UASs.

## Methods

The study was approved by the Ethics committee at the “Chaim Sheba” Medical Center, Israel (reference no. 4478-17). Medical files of patients undergoing ureteronephroscopy between 2013 and 2016 at our medical center were reviewed. During that period, 2601 endourologic procedures were overall performed. Patients undergoing RIRS for kidney stones with the use of UAS in an unstented ureter were included. Exclusion criteria included: prior impacted ureteral stones, presence of ureteral stones during surgery, prior ureteroscopies, prior ureteral drainage (double-J ureteral stent or PCN), documented ureteral strictures, prior radiation treatment, presence of renal or ureteral malignancy.

The pre-operative evaluation was done using NCCT or RUS. RIRSs were performed under general anesthesia by 3 fellowship-trained endourologists. Irrigation was done using 0.9% saline solution via a pressure-infusing system. After cystoscopy, a working guide-wire (hydrophilic 0.038-in. wire) is advanced up to the level of the renal pelvis. A semi-rigid 6.5/8.5fr URS is then carried out in order to inspect the ureter. After ruling out the presence of ureteral stones, a UAS is gently inserted up to the level of the proximal ureter under fluoroscopic guidance. If there is difficulty advancing the access sheath, ureteral dilation using serial dilators is carried out. Two sizes of UAS were used: COOK Medical Flexor 12/14Fr (wider) and 9.5/11.5Fr (narrower). The size of the UAS was determined at the surgeons’ discretion. We then perform a flexible ureteroscopy (using Storz Flex X2 7.5fr flexible ureteroscope) to inspect the proximal ureter, renal pelvis, and calyces for the presence of stones. Next, Holmium laser lithotripsy is carried out and/or stone extraction with a basket device. At the end of the procedure, a double pigtail ureteral stent is left for 5-14 days.

Follow-up includes laboratory tests (CBC, SMAC) and imaging (RUS after hydration), performed 2 months after stent removal and in each follow-up visit thereafter (follow-up regimen is tailored according to patient and stone characteristics). Hydronephrosis without ureteral stones was further evaluated by dynamic renal scan. Stone-free status was defined as no residual fragments, or the presence of residual fragments up to 3 mm, on renal ultrasound.

Demographic data (age, gender, BMI, systemic conditions), operative time, intra-operative complications (bleeding, perforation, incompletion of procedure) and clinical follow-up were collected.

The primary outcome of the study was to evaluate stricture formation rates. Strictures were suspected following a demonstration of new ipsilateral hydronephrosis with delayed secretion in a renal scan. Secondary outcomes included stone-free rates and development of flank pain, elevated serum creatinine level, and UTIs in the follow-up period. To evaluate the safety and efficacy of wider-caliber UAS, patients were stratified to 2 groups: wider (12/14Fr) and narrower (9.5/11.5Fr), in order to compare primary and secondary outcomes between the 2 groups.

Statistical analysis was performed using a chi-square test for nominal variables and student t-test for continuous variables. Multivariate analysis was performed using Binary logistic regression analysis for discrete dependent variables, and Linear regression analysis for continuous ones. The follow-up period was calculated from URS to an event, or to last follow-up visit.

Statistical significance was defined as *p* = 0.05. All reported *P* values are two-sided. Statistical analyses were performed using Statistical Package for Social Sciences (SPSS version 23.0, SPSS Inc.).

## Results

Demographic data, stone characteristics data and pre-operative evaluation are given in Table 1. Of the 165 patients included, on 86 a wide UAS was used, and on 79 a narrow one. The median age was 57 years with 62% males. The average number of stones treated was 1.5 and 1.6, while stone burden was 2.3 and 3.4 cm3, in the narrow and wide groups, respectively. The stone burden was significantly higher in the wider UAS group (*p* = 0.031). Pre-operative hydronephrosis was found to be a predictor of improvement in serum creatinine levels after surgery [0.02 vs. 0.09, β = − 0.09, 95% CI -0.17- (− 0.2), *p* = 0.039].

**Table 1:** Demographic and pre-operative data

UAS size	9.5/11.5fr	12/14fr	Total	*p*-value
N	86	79	165	
Gender (%male)	69.7	54.4	62.4	0.121
Median age (range)	53.5 (18-81)	58 (24-83)	57 (18-83)	**0.042**
BMI (median, IQR)	28.4 (24.5-32.3)	28.6 (25-31.2)	28.4 (24.7-31.8)	0.821
Systemic conditions				
Diabetes mellitus (%)	25.6	39.2	32.1	0.061
Hypertension (%)	41.9	48.1	44.8	0.424
Chronic renal failure (%)	7.0	3.8	5.5	0.299
Peripheral vascular disease (%)	1.2	0	0.6	0.339
Ischemic heart disease (%)	7.0	3.8	5.5	0.372
Hyperlipidemia (%)	30.2	40.5	35.2	0.169
Average number of conditions	1.4	1.52	1.45	0.534
Stone disease				
Number of stones (average, range)	1.5 (1-5)	1.6 (1-7)	1.6 (1-7)	0.506
Largest stone diameter (mm) (median, IQR)	13 (9-16)	15 (11-20)	15 (10-19)	**0.019**
Total stone burden (cm^3^) (median, IQR)	2.3 (1.0-5.4)	3.4 (1.6-8.0)	3.4 (1-7.4)	**0.031**
Lower pole stone (%)	39	38	38.7	0.907
Pre-operative hydronephrosis				0.461
No (%)	57.0	64.9	60.7	
Mild (%)	25.6	19.5	22.7	
Intermediate (%)	15.1	13	14.1	
Severe (%)	2.3	2.6	2.4	

*p*-value is for univariate analysis. Statistical significance was defined as *p* ≤ 0.05 and is shown in bold

A comparison of operative and post-operative complications between narrow and wide UASs is provided in Table 2. Length of stay was 0-1 day, depending on the time surgery was undertaken - when performed in the morning till noon, patients were discharged the same day. The rest were discharged the morning after surgery. Out of the 86 cases where a narrower UAS was used, in 13 (15%) a prior attempt of insertion of the wider UAS had failed. In 5 cases, serial ureteral dilators have been used before the introduction of a narrower UAS. There were no cases of failed insertion of UAS and no conversion to other methods of surgery. During a median follow-up period of 115 days, we did not observe any clinically significant stricture formation. Overall complication rate was 18.2%, including: flank pain (9%), UTI (2.4%), referrals to ER (12.1%). We did not observe any difference in complication rates comparing the two groups.

**Table 2:** Operative and post-operative complications and SFR

UAS size	9.5/11.5fr	12/14fr	Total	*p*-value
N	86	79	165	
Operative Time (median, minutes)	39.5	61	46.5	**0.002**
Intra-operative complications				
Bleeding	1	0	1	
Ureteral perforation	0	0	0	
Other	0	0	0	
Peri-operative complications				
Fever	1	5	6	0.077
Pain requiring IV/IM analgesics	4	6	10	0.429
Follow-up period (days) (median, IQR)	112 (88-187)	125 (82-237)	115 (82-216)	
Post-operative				
Flank pain (%)	8 (9.3%)	7 (8.8%)	15 (9.0%)	0.864
Creatinine change (mg/dl, median, IQR)	−0.03 (−0.18-0.03)	0.00 (−0.09-0.05)	−0.02(−0.12-0.05)	0.228
UTI (%)	3 (3.6%)	4 (5.1%)	7 (4.3%)	0.650
Referrals to ER	10 (11.6%)	10 (12.7%)	20 (12.1%)	0.906
Hydronephrosis (%)	0 (0%)	2 (2.5%)	2 (1.2%)	–
New hydronephrosis	0 (0%)	0 (0%)	0 (0%)	–
Stone-free-rate (%)	73.5%	85.7%	79.4%	0.056

*p*-value is for univariate analysis. Statistical significance was defined as *p* ≤ 0.05 and is shown in bold

Factors associated with SFR are presented in Table 3. The only factor which was significantly different between stone-free and non-stone-free patients was the number of stones treated (*p* = 0.003), and noteworthy is the fact that SFR was not affected by the total stone burden (*p* = 0.194).

**Table 3:** Comparison of Stone-Free and non-Stone-Free patients

Stone free status	Yes	No	Total	Univariate analysis	Multivariate analysis		
				*p*-value	*p*-value	OR	95% CI
N	127	33	160				
Male gender (%)	77 (60.6%)	23 (69.7%)	100 (62.5%)	0.338	0.761	1.15	0.45-2.96
Age (median, range)	58 (18-83)	56 (24-74)	57 (18-83)	0.407	0.188	1.02	0.98-1.05
Number of stones (median, range, mean, SD)	1 (1-7) 1.4 ± 0.9	2 (1-5) 2.0 ± 1.2	1 (1-7) 1.5 ± 1.0	**0.003**	**0.010**	**0.60**	**0.41-0.88**
Stone Burden (cm^3^) (median, IQR)	3.3 (1.0-6.3)	4.4 (1.2-8.0)	3.3 (1.0-7.2)	0.194	0.993	1.00	0.92-1.08
Pre-operative hydronephrosis (%)	47 (37%)	15 (45.4%)	62 (38.7%)	0.411	0.182	1.88	0.74-4.78
UAS size (% wide)	66 (51.9%)	11 (33.3%)	77 (48.1%)	0.056	0.191	1.87	0.73-4.78

Statistical significance was defined as *p* ≤ 0.05 and is shown in bold

When comparing the 2 sizes of UAS, wider UASs were used in cases of significantly larger stone burden, older patients and in those with more co-morbidities. In addition, there was a trend towards higher SFR with the use of wider UAS compared to the narrower one (85.7% vs. 73.5% respectively, *p* = 0.056). Moreover, wider UASs were not associated with higher rates of stricture formation or other complications (Table 4).

**Table 4:** Predictors of post-operative outcome after RIRS with the use of UAS

	Post-operative complications)Yes/No)			Creatinine change (mg/dL)		
	*p*-value	OR	95% CI	*p*-value	β	95% CI
Gender (Male/Female)	0.658	1.21	0.51-2.89	0.198	−0.04	−0.11 – 0.03
Age	0.217	0.98	0.94-1.01	0.161	− 0.002	− 0.005 – 0.001
No. of stones	**0.044**	**1.47**	**1.01-2.16**	0.741	−0.008	−0.04-0.02
Stone burden	0.171	1.04	0.98-1.10	0.165	0.004	−0.002 – 0.01
Pre-operative hydronephrosis (Yes/No)	0.138	0.51	0.21-1.23	**0.039**	**−0.09**	**− 0.17-(− 0.02)**
UAS size (Narrow/Wide)	0.780	1.13	0.47-2.69	0.380	−0.03	−0.10 - 0.40

*p*-value is for univariate analysis. Statistical significance was defined as *p* ≤ 0.05 and is shown in bold

## Discussion

Our study demonstrated two important issues regarding the use of UAS in unstented patients. First, wider UASs are safe, even among patients with virgin-ureters. In the whole series, we did not observe any case of post-operative ureteral stricture formation. Complication rates were low in the large-diameter UAS group, similar to the small-diameter UAS group. Second, despite handling larger stone burden, we found a trend towards higher SFR in the large-diameter UAS group.

There are several advantages to the use of UAS including improved visibility, easier removal of stone fragments, and decreased intra-renal pressures during the procedure. Large-diameter UASs emphasize these advantages [3–6]. Alongside these advantages of UAS, ureteral strictures may presumably complicate its use [5, 7, 8]. Earlier reports demonstrated ureteral injuries after UAS use, with a total incidence of up to 46%, including 13.4% of severe ureteral injuries [7].

Traxer et al. [7] evaluated the ureter endoscopically at the end of the procedure. At that time, the ureter does not have enough time to recover from the procedure. Accordingly, the rate of the observed injury has not been shown to predict future ureteral strictures. In a later report in an experimental animal model showed that after 2 weeks, only minimal inflammatory changes are evident [11], which can explain the absence of long-term effects of UAS.

Recent studies report lower rates of ureteral strictures after ureteroscopy. Tracy et al. [12] reported no long-term post-operative hydronephrosis after the use of UAS in 168 cases, and no difference in SFR and complication rates comparing between different UAS sizes. However, in 49% of the cases, a pre-operative stent has been placed. Similarly, Delvecchio et al. [9] reported post-operative stricture rate of 1.4% after 71 ureteroscopies, but with 32% of cases bearing a pre-operative ureteral stent. The presence of a ureteral stent prior to surgery can potentially prevent stricture formation by leaving a wider ureter for the UAS to enter and by that reducing wall ischemia. Therefore, it constitutes a significant bias when evaluating the post-operative stricture formation rates following the use of UASs.

There are several technical factors which are important to consider during URS in order to prevent ureteral damage. Unlike earlier reports [9], we do not use UAS for stones in the distal or mid-ureter. In addition, before introducing the UAS, we inspect the ureter endoscopically for the presence of ureteral stones. In cases that stones are identified, we treat them prior to the introduction of UAS to prevent ureteral trauma. Using these techniques, we demonstrated it is safe to use wider UAS even in virgin ureters.

In our study, we found a trend towards higher SFR in the wider UAS group even despite treating larger baseline stone burden. Stone-free patients compared to non-stone-free patients did not differ in age or gender but did differ in the number of stones (mean ± SD of 1.4 ± 0.9 Vs. 2.0 ± 1.2 respectively, *p* = 0.003) (Table 3). Interestingly, stone burden itself (summation of stone volumes) did not differ significantly (*p* = 0.194). Berquet G et al. [13] assessed SFR with and without the use of a UAS in 280 patients and reported that the use of UAS itself does not improve SFR.

Change in serum creatinine levels (before and after treatment) did not significantly differ between the two UAS groups, as the wider UAS did not compromise renal function. The change was found to be associated with pre-operative hydronephrosis (*p* = 0.039), which reflects the efficacy of the procedure in cases of pre-operative obstruction.

Intra-operative complications were very rare in our study: only 1 procedure in the narrower UAS group was stopped due to bleeding which impaired visualization. In the wider UAS group, no intra-operative complications were recorded. We also assessed post-operative complications for a median of 115 days after surgery: UTIs were very rare and occurred in only 4.3% of cases (3 patients in the small and 4 patients in the large-caliber groups, for a total of 7 patients), 3 of them had positive pre-operative cultures taken the day before surgery. In each UAS group, there were 10 ER visits mainly due to LUTS and stent pain (11.6% and 12.7% in the narrow and wide UAS groups, respectively), and there was no statistically significant difference between the two groups (*p* = 0.716). Operative time was significantly longer in the wider UAS group (39.5 Vs. 61 minutes, *p* = 0.002), which can be explained by the higher stone burden in that group. Despite the longer operative time (and the longer duration of UAS use), the rate of ureteral strictures was still low.

### Limitations

Our study is one of the first to assess stricture formation in previously unstented and unmanipulated ureters, though it is not without limitations. The series is retrospective in nature which may lead to selection bias. We tried to overcome this limitation by including all cases operated for kidney stones during the study period. The size of the cohort is relatively small and the follow-up periods are somewhat short, giving the rarity of the complication. The procedures were performed by 3 different surgeons, which may cause variation in technique, but on the other hand focuses on the method itself and not the skill of a specific surgeon. In addition, the choice of the size of the UAS was at the surgeons’ discretion and not randomly determined. This represents personal preferences of sheath size, but does not change the outcome – that UAS use is safe in unstented ureters. Ureteral dilation was carried out in 5 cases in which the ureter was thought to be tight, which by itself may possibly prevent stricture and cause bias. Nonetheless, we see ureteral dilation (if needed) to be an integral part of UAS use and should not be considered as an auxiliary procedure. Another drawback is the diagnosis or exclusion of stricture formation being indirect and based upon the appearance of new post-operative hydronephrosis with delayed ipsilateral drainage in renal scans, as we are interested in clinical significance. Indirect signs like hydronephrosis or hydroureter are accurately visible on RUS, although it is likely that estimated SFR is higher when using ultrasound in comparison to NCCT [10]. Nonetheless, in order to focus on the effect of UAS and prevent bias, many other factors that can influence ureteral strictures were excluded: pre-stenting, pre-nephrostomy, previous surgical manipulations, previous ESWL, and impacted ureteral stones. After excluding these factors, our results show that the clinical significance of injuries resulting from the use of UAS is very low, and ureteral strictures after the use of UAS in unstented patients are extremely rare, despite the usage of a wider UAS in 47.8% of cases. Therefore, when a more extensive lithotripsy is planned, it is safe in our opinion to use a large-caliber UAS to improve visualization and enable extraction of larger fragments.

## Conclusions

To conclude, these data suggest that the use of UAS during RIRS in an unstented ureter is safe and is not associated with increased risk for ureteral stricture formation. Furthermore, the use of a wider UAS was not found to be associated with higher complications rate. Due to its observed rarity, large scale randomized prospective trials should be conducted to elucidate the real magnitude of ureteral strictures after the use of UAS and the effect of different sizes of UAS.

## Data Availability

The data that support the findings of this study are available on request from the corresponding author A.S. The data are not publicly available due to them containing information that could compromise research participant privacy.

## Abbreviations

ER: Emergency room
ESWL: Extra-corporal shock wave lithotripsy
NCCT: Non-contrast computerized tomography
RIRS: Retrograde intra-renal surgery
SFR: Stone-free rate
UAS: Ureteral access sheath
URS: Ureteroscopy
RUS: Renal Ultrasound
UTI: Urinary tract infection
